# Development of Items Designed to Evaluate Activity Performance and Participation in Children and Adolescents with Spinal Cord Injury

**DOI:** 10.1155/2009/854904

**Published:** 2009-10-25

**Authors:** Christina L. Calhoun, Stephen M. Haley, Anne Riley, Lawrence C. Vogel, Craig M. McDonald, M. J. Mulcahey

**Affiliations:** ^1^Shriners Hospitals for Children-Philadelphia, 3551 North Broad Street, PA 19140, USA; ^2^Health and Disability Research Institute, School of Public Health, Boston University, 715 Albany St., T5W, Boston, MA 02118, USA; ^3^Deparment of Population, Family and Reproductive Health, Bloomberg School of Public Health, John Hopkins University, 615 N Wolfe Street, Rooom E4539, Baltimore, MD 21205, USA; ^4^Shriners Hospitals for Children-Chicago, 2211 North Oak Park Ave, Chicago, IL 60707, USA; ^5^Shriners Hospitals for Children-Northern California, 2425 Stockton Blvd., Sacramento, CA 95817, USA

## Abstract

*Background/Objective*. Outcomes-based data, whether used clinically or for research, are difficult to collect in the pediatric spinal cord injury (SCI) population due to a lack of appropriate assessment measures. The purpose of this paper is twofold: to describe the process by which two item pools were developed to evaluate activity performance and participation among children with SCI and to introduce the resultant items specific to pediatric SCI. *Methods*. The process of item development, including construct development, review of related assessment tools, chart review, item writing and refinement using focus groups, cognitive interviews, and further refinement, was used to create the items pools for activity and participation for children and adolescents with SCI. *Results*. A total of 347 items were written for the activity performance construct and 61 items were written for the participation construct. Several domains were established within each construct and items were written for both child and parent respondents. *Conclusion*. The process of detailed item development is the first step in the process of developing an outcomes instrument for children and adolescents with SCI to assess activity performance and participation. The items are representative of pediatric SCI because they address areas specific to children and adolescents with SCI such as wheeled mobility, upper extremity function with adaptive equipment, role performance, and socialization. After testing these items in calibration studies, we will determine if these items can be developed into effective computer-adaptive testing applications.

## 1. Introduction

Collecting routine functional outcomes in children with spinal cord injury (SCI) has significant practical implications, as health care providers, social agencies, and school systems have a need to know if children are progressing, regressing, or maintaining their functional levels. The ability to monitor children over time is essential to ensure that children and adolescents with SCI are meeting developmental milestones and achieving social role expectations, and to assist with determining the need for services from rehabilitation facilities and community and education-based agencies [1].

In the current environment, outcomes-based data are difficult to collect in the pediatric SCI population due to a lack of appropriate assessment measures. Assessments for adults with SCI are available and often used clinically; however they may be developmentally inappropriate for children. Although instruments are available to assess function in children, they fall short in adequately providing an understanding about daily functioning with an SCI, such as mobility via a wheelchair or use of a hand splint, role performance, like household chores and school work, and socialization of children with SCI.

The Functional Independence Measure (FIM) [2] is the most commonly used instrument to evaluate what individuals over the age of 7 years can do after SCI, despite the many documented limitations. Substantial ceiling and floor effects have been reported with the FIM for adolescent and adult SCI samples, particularly with long-term follow-up. Hall et al. [3] report that 86% of patients with tetraplegia have floor effects (lowest possible score) at hospital admission on the motor FIM. Even more striking, nearly 36% of patient with paraplegia have a ceiling effect (highest possible score) at rehabilitation hospital discharge, and 75% of patients with paraplegia have a ceiling effect on the motor FIM at 3-years post-SCI. In addition, the FIM was noted to have limitations in detecting clinically meaningful changes in a series of children with SCI. As was reported by Garcia et al. [4], the WeeFIM [5], the FIM for children and the FIM may be insensitive to certain clinically important performance changes. For example, a child with paraplegia admitted to a rehabilitation hospital with independent manual wheelchair propulsion, and subsequently discharged as independent in ambulation with an assistive device, will not have an improved score on the FIM, because both methods of mobility are given the same score (modified independence in mobility). In our own published work [6], we demonstrated that the FIM was insensitive to clinically meaningful changes following upper extremity tendon transfers in children with SCI.

The Craig Handicap Assessment and Reporting Technique (CHART) [7] is a popular measure of participation in SCI programs but contains developmentally inappropriate items for children and adolescents [8]. The Canadian Occupational Performance Measure (COPM) [9, 10], a tool that measures changes in client-perceived performance of self-identified goals, does not produce a composite score of activity performance that is comparable longitudinally and across populations but can provide an understanding about activity performance at a point in time in a child's life [11]. Standardized self-report QOL measures for children have not been shown to be appropriate for clinical research or long-term monitoring of children with SCI. The Pediatric Outcomes Data Collection Instrument (PODCI) [12], Pediatric Quality of Life Inventory (PedsQL) [13], and CHQ [14] have limited usefulness for children and adolescents who use wheeled mobility due to specific wording and inappropriate items, such as “walking a mile” or “standing at a sink.” The Children's Assessment of Participation and Enjoyment (CAPE) [15] is a relatively new measure of participation for children. Based on our experience, the CAPE has a high response burden and does not contain participation items important to children with SCI such as participation in their own self care and participation in organized school activities. Clearly, the development of a targeted pediatric SCI measure should have a large set of items to cover the wide set of functional abilities and ages in this population and have content that is specific to some of the unique functional tasks that children and adolescents with SCI encounter.

Contemporary measurement approaches such as Item Response Theory (IRT) methods provide a promising means to achieve psychometrically adequate, comprehensive, and precise outcome instruments that are practical for widespread application in clinical and research settings. IRT is a set of statistical models for the analysis of multiple categorical variables that measure the same concept (such as a content domain within a parent or child survey). There is intense worldwide interest in using IRT methods to foster the next generation of practical and precise instruments for monitoring health care outcomes [16–19], while overcoming the chronic breadth, precision, and practicality challenges of traditional outcome instruments. A contemporary method of creating new instruments is to develop large item pools and then calibrate them into item banks that can then be used to support the development of computer-adaptive testing (CAT).

Computer adaptive testing programs utilize extensive item banks, available for administration, but any one respondent is only provided to the items optimal for their abilities. Each CAT administration is adapted to the unique ability level of each respondent. An adaptive test first asks questions in the middle of the ability range and then directs questions to an appropriate level based on the individual's responses. This allows for fewer items to be administered, while gaining precise information regarding an individual's placement along a continuum of ability or health status. We have recently demonstrated the feasibility of building CAT platforms for a successful clinical trial for children with Lysosomal storage disease [20], for monitoring children enrolled in inpatient and outpatient physical rehabilitation programs [21], and for evaluating children at the point-of-care in a busy orthopedic spine practice [22]. CAT has also been successfully applied using the FIM items showing that CAT can be used to reduce data collection with negligible reduction in precision [23].

The quality and relevance of items is a very important factor in the success of functional outcome measures in children with SCI. Particularly with a CAT, in which a limited number of items are administered to each individual, the choice and clarity of items is critical to the performance of CAT.

As a direct response to the void of an appropriate outcomes instrument for use with children with SCI, we have developed large item pools to evaluate activity performance and participation. These item pools will be further assessed and eventually be tested by getting responses to the items from a large sample of children and parents. Once tested, a final set of items (item banks) will be assembled for use in the CAT [24]. Our effort in development of item banks for eventual CAT platforms represents unique contributions to the field of pediatric SCI rehabilitation and measurement in two ways. First, our items are specific to SCI and have been designed to evaluate actual activity performance and participation at home, school, and the community environments. Secondly, we have established items and written response scales to obtain both parent and child reported outcomes. In this way, we plan to contribute a parent and child reported outcome measure of activity performance and participation for children with SCI that uses 21st century CAT technology thereby minimizing response burden but providing precision in measurement.

The purpose of this paper is twofold: to describe the process by which two item pools used to evaluate activity performance and participation among children with SCI were developed and to introduce the resultant items specific to pediatric SCI.

## 2. Methods

The development of the item pools began by agreeing on the conceptual definitions for two constructs, activity performance and participation. *Activity performance is defined as children's execution of complex functions; these functions represent specific tasks that can be done in isolation of others or with others. Activity considers ease, level of independence, and quality of execution of specific tasks. It represents the individual perspective of function. Participation is defined as children's involvement in life situations across physical, social, spiritual, and virtual environments including home, school, and community*. The conceptualization and definitions of the constructs are consistent with those of the World Health Organization [25], The Washington Group on Disability Statistics [26], the ICF Model [27], the conceptual model of the disability creation process [28], the conceptual model described by King et al. [29], and the Commission on Practice, Occupational Therapy Practice Framework [30]. An underlying assumption of the constructs is that capability and capacity for activity inform (but do not fully predict) participation. Limitations in activity performance place one at a disadvantage for participation.

**Figure 1:**
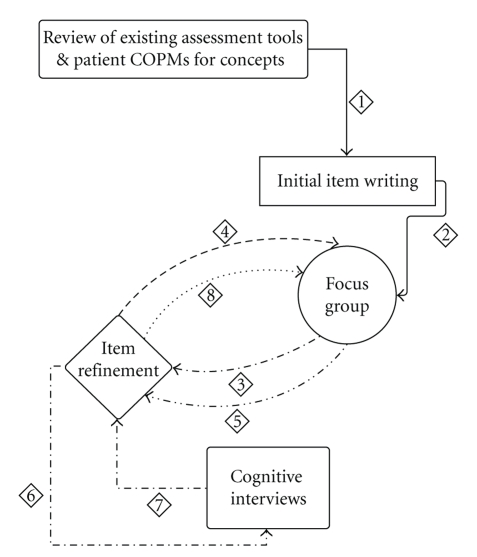
Item development.

For each construct of activity performance and participation, domains were established. For activity performance, the domains include self care, children's areas of occupational performance, and mobility. Self care involves activities of daily living, such as feeding, dressing, and hygiene; children's areas of occupational performance include typical routines that children engage in such as schoolwork, chores, leisure, and play. Mobility includes activities such as transitions, transfers, and moving about using various modes such as power or manual wheelchair use or ambulation. Participation is evaluated based on an internal perspective (self) and on an external perspective (compared with peers). For every activity and participation item developed, a child respondent version as well as a parent respondent version was written. Item development was done with an iterative process detailed in Figure 1.

**Item Development**

The first step was to review 24 outcome measures commonly used clinically to evaluate physical functioning, participation, and quality of life in the pediatric population (Table 1). Tasks or concepts included in these measures and deemed relevant to the pediatric SCI population were organized according to domain so that items could be written for that task or concept.

In addition to review of existing outcomes measures, we conducted a review of patient medical records to obtain patient-identified goals for rehabilitation that were generated from administration of the Canadian Occupational Performance Measure (COPM). The COPM uses an individualized client-centered approach, allowing therapists to evaluate change in a patient's self perceived performance as a result of an intervention [9]. For the last 10 years, our rehabilitation program has used the COPM as a primary rehabilitation tool with children with SCI; these assessments provided us activity performance and participation goals that were identified by children with SCI. These COPM goals were also organized according to domain so that items could be written for that goal. Some common goals generated as a result of the COPM included *putting a cd into the cd player, turning a page in a newspaper/magazine, tossing a ball, and playing video games*.

The Delphi technique, a qualitative data collection method in which a group of people are come together to brainstorm ideas related to a key issue [31], was used to identify, refine, and write items for the activity performance and participation constructs. The team of professionals involved in this process included a pediatrician, a physiatrist, and an orthopedic surgeon, 3 nurses, 2 psychologists, a social worker, 5 occupational, 5 physical, and 2 recreational therapists, and 1 speech therapist; all have extensive experience in the treatment of pediatric SCI. This team met for 3 separate in-person meetings. These meetings were for initial item development, item refinement, and final item consensus. The initial item development focus group took the tasks or concept from established assessment tools and COPM goals and wrote the first draft of items for each domain.

Following the initial item development focus group, a subgroup of 4 individuals (2 physical and 2 occupational therapists) took the initial items or important concepts previously established and wrote additional items for each domain. This was done to address gaps identified by the Delphi technique. In addition to writing each item, this group developed an intent for each item. The intent was clarified in order to ensure each item included only one concept.

For the 2nd focus group meeting, the first phase of item refinement, all written items and intents were placed on individual index cards. Items within each domain were arranged in an order of difficulty that was thought to be the easiest to the hardest. Focus group participants read each item and intent and commented on writing style and wording, the easiest to the hardest order, whether the item and intent matched, and how well they thought the item applied. Within the large group, this input was used to come to a consensus on each individual item.

In order to truly solicit feedback from children and adolescents the next phase of item development included cognitive interviews. The purpose of a cognitive interview is to determine the readability, comprehension, and meaning of a questionnaire item [32]. A small team of physical, occupational, recreational, and speech therapists were trained in conducting cognitive interviews. Each item was asked to children ages 7–18 with SCI and to parents of children with SCI. Notes were taken by the interviewer during the interview, and the recorded interviews were transcribed for later review. Based on the cognitive interview notes and transcriptions, items were continuously refined as the interviews were conducted and reviewed for problems. Each problematic item, prior to additional refinement, was coded based on the type of issue children or parents had with the item.

The refined items and their intents were again placed on individual index cards. A final consensus focus group meeting occurred in order to give any final feedback and narrow the total number of items for each domain. These items were again reviewed by 2 physical therapists and 1 occupational therapist and final refinements were made.

## 3. Results

**Figure 2:**
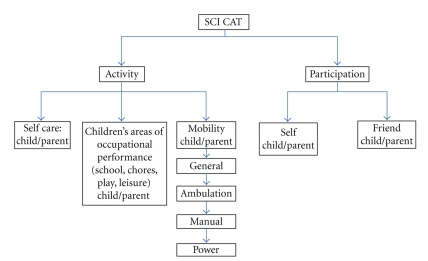
SCI CAT constructs, domains, and subdomains.

This iterative item development process resulted in a pool of 347 items for the activity performance and participation constructs each with domains and some subdomains (Figure 2).

**Cognitive Interviews**

A total of 33 child subjects and 13 parent subjects participated in the cognitive interview process. The children ranged from age 7 to 18; their grade in school was not considered in the inclusion criteria. The coding method used to modify and refine items is detailed in Table 2. Two important outcomes of the cognitive interviews with regards to a child's ability to read and comprehend items were related to age and grade. Children under age 8 had many items which were commented on by interviewer and coded as unable to read or difficulty with comprehension. In addition, several children who completed interviews were in kindergarten, first or second grades. These children also had difficulty with reading and comprehension. Therefore it was decided that the refined items were written for children 8 years and older who have completed second grade.

**Item Development**

The final number of items developed for each and examples are provided in Table 3. Items for child and parent respondents differ only in terms of how the person is referred to, for example, “*I can turn my power wheelchair on*” and “*My child can turn the power wheelchair on*.”

Because adequate items to assess the pediatric SCI population do not exist in current outcomes measures, new items were often written, making the task appropriate for and specific to both pediatrics and spinal cord injury. Within the mobility domain of activity performance, 4 subdomains were generated. The first was general mobility; the items in this domain focused on actions such as bed mobility and transfers. These items were extremely specific, for example, asking about not only an individual's ability to move in bed (i.e., supine to sit) but also the ability to get under the sheets in bed once in bed. Two subdomains were created regarding the use of wheeled mobility: power or manual wheelchair use. These items, regardless of wheelchair type, not only addressed an individual's ability to maneuver the wheelchair but also addressed the terrain, whether they need to carry something in addition to moving the wheelchair and wheelchair parts management. Lastly, while ambulation is a less used means of mobility in the SCI population, items were written to address ambulation with various assistive device, and on various terrains.

A second domain within activity performance was self care. This domain addressed tasks such as feeding, dressing, and hygiene. Items were written to be sensitive enough to distinguish between various levels of upper extremity ability. These items included the use of hands splints if applicable, completing an activity with two hands, one hand, or even an individual's mouth. The third domain within activity performance is children's areas of occupational performance. These items addressed play and leisure, chores and household tasks, and school performance. Like self care, these items were written to distinguish between various levels of upper extremity function and asked questions using hand splints and whether a task is completed with two hands, one hand, or with an individual's mouth.

The second construct addressed in the item pool is participation, which contained two domains: self and friend. Items were written to address participation in various places, such as the home, school, and community environments, and with various people such as family and friends.

## 4. Discussion

The process of item development detailed here represents the first step in the development of item pools for an eventual CAT platform for children and adolescents with SCI to assess activity performance and participation. The items developed are truly representative of the activities necessary for an individual with SCI to function but often not assessed in other tools. In addition, to avoid ceiling and floor effects, the participation items were written to include items ranging from those completed in a home setting to those that require transportation or financial support.

The iterative process used for item development models the methodology used by others who have also developed item pools and outcomes assessments [33–36]. Content validity of the item banks by evidenced the expertise of the item writers, the use of COPM goals, and by using direct and indirect patient feedback. The team of healthcare professionals who wrote the items all had extensive experience in the treatment of SCI. The multidisciplinary approach to item writing ensured that many points of view from each discipline were considered in the writing of each item. In addition, using a team approach expanded the range of capabilities included in the items, ensuring that there are appropriate items for all levels. An additional strength of this process included the use of patient self reported goals (COPM) and patient and parent input from the cognitive interviews. Because the COPM goals are client directed, we were able to directly obtain concepts important to children with SCI thereby further establishing face validity. The cognitive interview process allowed for direct feedback from this population regarding their interpretation of the item. Items were modified, clarified, and simplified based on this feedback. In some instances the entire item was removed from the pool because while the team writing that the item thought it may be important, the child or parent respondent simply did not do it.

Further work is necessary to complete the process of establishing items banks. This includes a study to determine if the item pools calibrate into item banks that can be used to support CAT. Our eventual CAT assessment tool will represent the first outcome measure designed specifically for children with SCI.

## Figures and Tables

**Table 1:** Assessment tools.

Assessment of Life Habits
Children's Assessment of Participation and Employment (CAPE)
Craig Handicap Assessment and Reporting Technique (CHART)
Developmental Assessment of Young Children (DAYC)
Canadian Occupational Performance Measure (COPM)
Child Health Questionnaire (CHQ)
Facilitators and Barriers Survey/Mobility (FABS)
Functional Independence Measure (FIM)
Gross Motor Function Measure (GMFM)
Hawaii Early Learning Profile (HELP)
The Kohlman Evaluation of Living Skills (KELSs)
Movement Assessment Battery for Children (MABC)
Pediatric Activity Card Sort (PACS)
Participation Survey/Mobility (PARTS)
Peabody Developmental Motor Scales (PDMSs-2)
Pediatric Evaluation of Disability Inventory (PEDI)
Pediatric Quality of Life Inventory (PedsQL)
Pediatric Outcomes Data Collection Instrument (PODCI)
School Function Assessment (SFA)
Sensory Profile (Infant/Toddler)
Shriners Pediatric Instrument for Neuromuscular Scoliosis (SPINS)
Social Skills Rating System
Toddler and Infant Motor Evaluation (TIME)
WeeFIM System

**Table 2:** Cognitive interview coding for problematic items.

Symbol	Meaning
C	Comprehension
R	Unable to read
D	Recommended a different word choice
S	Poor specificity
E	Cannot answer because child does not do
A	Accessibility issue

**Table 3:** Total number of items and examples for each construct, domain and subdomain.

Construct	Domain	Subdomain	Number of items	Child respondent examples	Parent respondent examples
Activity performance	Mobility	General mobility	25	- When sitting, I can turn my head from one side to another. - I can sit on the edge of my bed.	- When sitting, my child can turn his or her head from one side to another. - My child can sit on the edge of the bed.

		Power mobility	24	- I can turn my power wheelchair on. - I can move my power wheelchair in a busy hallway with a lot of people.	- My child can turn the power wheelchair on. - My child can move the power wheelchair in a busy hallway with a lot of people.

		Manual mobility	44	- I can stop my manual wheelchair. - I can push my manual wheelchair over a small bump in the floor.	- My child can stop the manual wheelchair. - My child can push the manual wheelchair over a small bump in the floor.

		Ambulation	58	- Using crutches, I can walk on grass outside. - I can step up a curb.	- Using crutches, my child can walk on grass outside. - My child can step up a curb.

Activity Performance	Self care		98	- With my splint I can unzip my jacket. - Including fixing my clothes, set up and clean up, I can complete my bowel program. - I can feed myself soup with a spoon	- With my splint my child can unzip a jacket. - Including fixing clothes, set up and clean up, my child can complete his or her bowel program. - My child can feed himself or herself soup with a spoon.

Activity performance	Children's areas of occupational performance		98	- I can make my bed. - Using only one hand, I can use the videogame controller. - I can take a book out of my bookbag.	- My child can make the bed. - Using only one hand, my child can use the videogame controller. - My child can take a book out of a bookbag.

Participation	Self and friend		61	- At home I listen to music. - I go places with my family. - I eat lunch with my friends in the school cafeteria. - I try clothes on at the store.	- At home my child listens to music. - My child goes places with the family. - My child eats lunch with friends in the school cafeteria. - My child tries clothes on at the store.
